# Municipal Versus State Control of Tuberculosis1Read at the November meeting of the Veterinary Medical Association of New York County.

**Published:** 1899-02

**Authors:** Edwin B. Ackerman

**Affiliations:** Borough of Brooklyn, N. Y.


### MUNICIPAL *VERSUS* STATE CONTROL OF TUBERCULOSIS.[1]

By Edwin B. Ackerman, D.V.S.,
BOROUGH OF BROOKLYN, N. Y.

In opening this discussion it will be necessary for me to quote the State laws and municipal ordinances covering this question.

Chapter 661, law 93, was made under an appropriation to kill tuberculous cows, but without appraisal of cattle. This was amended by Chapter 664, law 94. Under these laws the following regulations are laid down in Sections 60 to 65 of the *Public Health Laws*, all counties, State of New York:

### *Tuberculosis and Glanders.*

Section 60. Jurisdiction of State Board.

Sec. 61. Suppression of tuberculosis.

Sec. 62. Destruction of domestic animals affected with tuberculosis or glanders.

Sec. 63. Compensation to owners.

Sec. 64. Penalties.

Sec. 65. Special Committee of State Board.

[1] Read at the November meeting of the Veterinary Medical Association of New York County.

SECTION 60. *Jurisdiction of State Board.* The State Board of Health shall investigate concerning the existence and cause of tuberculosis in cattle and the danger to the public health therefrom, and shall use all reasonable means for averting and suppressing such disease. Such Board may cause all proper information in its possession respecting tuberculosis in cattle to be sent to the local Board of Health nearest to the cattle affected, and may add thereto such useful suggestions as to the removal of the sources of danger therefrom, or as to the destruction of such cattle, as such Board may deem proper. The local health authorities shall supply to the State Board of Health like informations and suggestions respecting the existence of tuberculosis in cattle.

SEC. 61. *Suppression of tuberculosis.* Whenever tuberculosis shall be found among cattle in any part of the State the State Board of Health shall take measures to suppress such disease and prevent the spread thereof, and may order all persons to take such precautions against the spread of such disease as it may deem necessary or expedient. Such Board may call upon any peace officer in the neighborhood of such disease to enforce the orders of such Board respecting such disease, and to observe and carry out the rules, orders, and instructions which he may receive therefrom. Such Board may prescribe regulations for the destruction of cattle affected with tuberculosis, for the proper disposition of their hides and carcasses and of all objects which might convey the infection or contagion, and for the disinfection of premises, buildings, boats, cars, stables, and other objects or places from or by which such infection or contagion might be communicated. The State Board of Health may employ such medical aid, veterinary practitioners, and other persons as it may deem necessary, to assist in the inspection, isolation, destruction, or disposition of cattle affected with tuberculosis, prescribe rules and regulations for such inspector and employés, and fix their compensation.

SEC. 62. *Destruction of domestic animals affected with tuberculosis or glanders.* Whenever the State Board of Health may deem it necessary for the prevention of the spread of tuberculosis in cattle, such Board may cause to be killed any animal affected thereby, or which, by contact with diseased animals or by exposure or infection or contagion therefrom, such Board may determine is liable to contract or communicate such disease; but no such diseased animal shall be so killed on account of tuberculosis unless first examined by a veterinary practitioner in the employ of the State Board of Health, and, if desired by the owner, appraised as hereinafter provided. A local Board of Health shall, pursuant to rules and regulations prescribed by the State Board of Health, cause to be killed every horse affected with glanders found within its jurisdiction; but no horse shall be so killed on account of glanders until the value thereof be appraised as hereinafter provided.

SEC. 63. *Compensation to owners.* To determine the value of such animal the comptroller shall designate some competent, disinterested person, residing within the judicial district in which such animal may be, to act as appraiser, with an appraiser to be selected by the owner of such animal, who shall promptly fix a time when they shall view such animal, and shall proceed to appraise the value thereof. In case of a disagreement between the two appraisers, the third appraiser shall be selected by them, and the estimate of the value of either two of them shall be final. The animal shall be appraised at its sound value, provided, however, no single, unregistered animal shall be appraised at more than $60. Each appraisal shall be in writing, signed by the appraiser or appraisers agreeing, and shall be delivered by them, if the animal be suspected of tuberculosis, to the veterinary practitioner in charge of such animal; and if the animal be a horse affected with glanders, to the Secretary of the local Board of Health having jurisdiction thereof. Upon the delivery of such appraisal such animal shall be killed, as hereinbefore provided; and if it be killed on account of tuberculosis the veterinary practitioner in charge thereof shall forthwith make a post-mortem examination of the animal, and if it shall be discovered upon such post-mortem examination that the animal was affected by tuberculosis, the owner of the animal shall be entitled to receive one-half of the appraised value; provided, however, that not more than $60 shall be paid for a diseased, registered animal, and not more than $25 shall be paid for a diseased, unregistered animal; but if such examination of the animal killed on account of tuberculosis discloses that the animal was not affected with tuberculosis, the owner shall be entitled to receive the full appraised value. The written appraisal of the value of an animal killed on account of tuberculosis, and a written statement of the result of the post-mortem examination thereof, signed by the veterinary practitioner in charge thereof, shall forthwith be transmitted by such veterinary practitioner to the Secretary of the State Board of Health, who shall file the same in his office. The Secretary of the local Board of Health having jurisdiction in the case of a horse affected with glanders shall, in case such horse is killed, upon receipt of the written appraisal, signed by the appraiser or appraisers, as hereinbefore provided, forthwith make and sign a certificate of such fact, and transmit such appraisal and certificate to the Secretary of the State Board of Health, who shall file the same in his office. Upon receipt from the veterinary practitioner in the case of an animal killed on account of tuberculosis, or from the Secretary of the local Board of Health having jurisdiction in the case of a horse killed on account of glanders, such Secretary of the State Board of Health shall forthwith make a written certificate, signed by him, setting forth the name and post-office address of the owner of the animal killed, and the amount which such owner is entitled to be paid on account of the killing of such animal, and shall forthwith transmit such certificate to the comptroller, who shall issue his warrant upon the treasurer for the payment to such person of the amount so certified, and shall mail the same to such person at his post-office address as it appears by such certificate. No compensation shall be allowed to any person who shall have wilfully concealed the existence of tuberculosis or glanders among his animals, or upon his premises, or who, directly or indirectly, by act or wilful neglect, shall have contributed to the spread of such diseases or either of them; and no compensation shall be made under the provisions of this act to any owner for animals killed unless the animal or animals killed shall have been actually owned and possessed by the owner thereof within this State for a period of three months prior to such condemnation. The appraisers to be appointed as aforesaid by the Comptroller, shall hold office during the pleasure of the State Board of Health. Each appraiser appointed shall receive as compensation the sum of $5 per day for each day actually employed, and shall also be paid his actual necessary disbursements; but no claim for services or disbursements shall be allowed or paid unless accompanied by a verified detailed statement thereof.

SEC. 64. *Penalties.* Any person refusing to obey or violating an order, rule, or regulation of the State Board of Health respecting tuberculosis in cattle, adopted pursuant to law, shall be liable to a penalty of $100, recoverable by the State Board of Health and applicable to the payment of the expenses of such Board in carrying out the provisions of this article.

SEC. 65. *Special committee of State Board.* The State Board of Health may appoint two of its members as a committee, whose particular duties shall be to carry out the provisions of the public health law relating to tuberculosis in cattle, and such members so appointed shall be entitled to receive a salary of $250 per month and any necessary expenses, and they shall hold office for one year. Such committee shall keep a complete record of all work done and submit monthly reports thereof to the State Board of Health.

*The Sanitary Ordinances of the City of New York* are as follows, viz.:

Section 31 of the sanitary regulations reads as follows: That no cattle shall be killed for human food while in a diseased condition, and all such diseased cattle in the City of New York and the place where found, and their disease, shall be at once reported to the Department of Health by the owner or custodian thereof, that the proper order may be made relative thereto or for the removal thereof from the city.

SEC. 120. That no diseased cattle or other animals, nor any that have been exposed to any disease that is contagious among such animals, shall be brought into the City of New York.

SEC. 121. That no animal having glanders or farcy, or any contagious disease, or that shall die thereof, shall be removed, disposed of, or exposed in any street or public place in said city without a written permit from said Board of Health, and then only in accordance with the terms of such permit.

SEC. 185. That every veterinary surgeon who is called to examine or professionally attend any animal within the City of New York having glanders or farcy, or any contagious disease, shall, within twenty-four hours thereafter, report in writing to the Board of Health of such city the following facts, viz.: 1. A statement of the location of such diseased animal. 2. The name and address of the owner thereof. 3. The type and character of the disease. This compels the report on all cases of tuberculosis.

SEC. 59. That no person shall sell, deliver, or offer or have for sale or keep for use or bring or send to said city any skimmed, watered, or adulterated milk, or milk known as "swill-milk," or milk from sick or diseased cows.

Sections 75, 76, 77, and 78. Driving and leading cattle through the streets. These sections prohibit the driving or leading of cattle through the streets except under certain conditions (as one person for each cow, animals properly marked for identification of owner or destination, by sign or symbol, which sign or symbol, etc., must be filed with the Department of Health when applying for permit), and then only by permission of the Department of Health.

Now to review the law. By State laws and regulations all parts of the State should and would receive equal inspection and care, and all dairymen would receive proper compensation for their diseased animals. This would be fair and just, and the people would be protected at their own expense for a pure, healthy article of food; the farmer would get rid of his diseased cows at a small proportion of the loss, and he would have healthy herds with which to put his produce upon the market. This to be done fair would require an appropriation large enough to carry on the work, and in all counties, and not in a few favored counties as heretofore. Under tubercular inspection by city ordinances everything is done by coercion. A dairyman is notified that on such a day his herd will be tested with tuberculin. If he resist or refuses to have his cows tested, he is arrested on the charge of keeping cows without a permit, and his stable is immediately quarantined, thus shutting up his business.

You will notice that Section 120 reads that no diseased cattle shall be brought into the city of New York. Thus if the stable door was kept locked the dairymen of our city would be protected and not get diseased cows in their herds. Now, then, as the stable door is open, and cows with tuberculosis are allowed in, our dairymen get these cows, disease and all, in their dairies. The Health Department then comes along and confiscates them on a tuberculin test under their unlimited power and authority. This is all right as far as it goes, but under Section 31, if they have a private test made they or their attending veterinarian are required to report same to the Health Department, and thus they would lose them. If, on the other hand, such cases are not reported, and the owner tries to dispose of same, he is confronted by Sections 75 to 78, which prohibits him from leading or driving cows through the streets without a permit from the Health Department. He is thus allowed to buy cows with the disease, but is hampered from disposing of them to any advantage, no matter how slight the animal is affected.

His loss becomes total, because when the Health Department condemns a cow they take the whole carcass, unless the owner will sign a release, in which case he can have the hides. While the regulations of our Bureau of Animal Industry and the conclusions of our National Veterinary Association, are that in localized tuberculosis the meat may be used for food, while generalized tuberculosis should be condemned, and as a good proportion of the cows condemned under the tuberculin test have only a small localized lesion, then carcass, skin, etc., could be utilized to the owner's advantage.

Of course this test is made largely in dairies for the protection of the milk-supply. But under our municipal ordinances this inspection is confined to the dairies located in the city limits, and only protects the milk supplied by them, and as the city supply from local dairies is less than one-twentieth of the entire quantity used, the protection is a failure, to the competitive disadvantage of our local dairies, and to the advantage of suburban or country milkmen, thus making the competition unfair. In other words, we rid the local dairies of tuberculous cows, but we do not make our general milk supply any better protected. It therefore becomes self-evident and more fitting that this disease should be controlled by the State laws and regulations, with the aid and inspection of the various local health departments, in this way.

To-day I understand that animals coming into this State are tested with the tuberculin test, and many of those that fail to react are shipped to Pennsylvania and Massachusetts, because they will not receive animals except they will pass the test, while those cattle they do not want and those that are not tested at all are sold in this State. Thus the cow-stable door is wide open. So if the State began in Buffalo and kept tuberculous cows out, it would be an excellent start. Then by a systematic inspection of rural districts our out-of-town dairies supplying milk to our large cities would be controlled; that would be two forms of inspection. Then, as an extra precaution, our cities should establish a quarantine-station for all cows shipped into the city, and all cows with State certificate or tag could either be tested or passed, while State cows shipped into the cities could be detained for a test. This would shut the door against the disease all along the line, and then to lock them the city could make the local inspection or test in our city dairies, and there catch anything that had escaped detection or acquired the disease in the dairies, and at the same time our local Health Department should be given the privilege of making inspection on State authority or with a State inspector upon any herd of cattle whose milk was sent to our city.

To conclude, all district inspectors, quarantine inspectors, and local departments of health could report all cases of tuberculosis to the State authorities, whether that be the State Board of Health, the Department of Agriculture, Bureau of Animal Industry, Board of Tuberculosis Commissions, or a State veterinarian, for appraisal, so that owners might be indemnified up to a certain value, for example, as fixed by the present law.

It remains with this body, our State Veterinary Medical Association, the Dairymen's Association of all the counties of Greater New York, and our city Department of Health to use every means in their power, individually and collectively, to see to it that some proper measure is prepared or law passed by our next Legislature to rid our herds of tuberculous cows on an equal basis with our rural dairymen in competition with our local dairies, and to the advantage of all our milk, so that we may get a healthy food.

At some future time, gentlemen, I will have something to say on hygienic marketing of milk.

